# Supplementary material for: Association between oral microbiome and seven types of cancers in East Asian population: a two-sample Mendelian randomization analysis
Source: Front Mol Biosci. 2023 Nov 21;10:1327893. doi: 10.3389/fmolb.2023.1327893 (PMC10702768; doi:10.3389/fmolb.2023.1327893)
Supplement: Supplementary file 1 [file DataSheet1.ZIP › Supplementary Materials/MR plots of saliva/saliva═╝/Prostate cancer/pheno.2386_to_prostate cancer_leave_one_out.pdf]

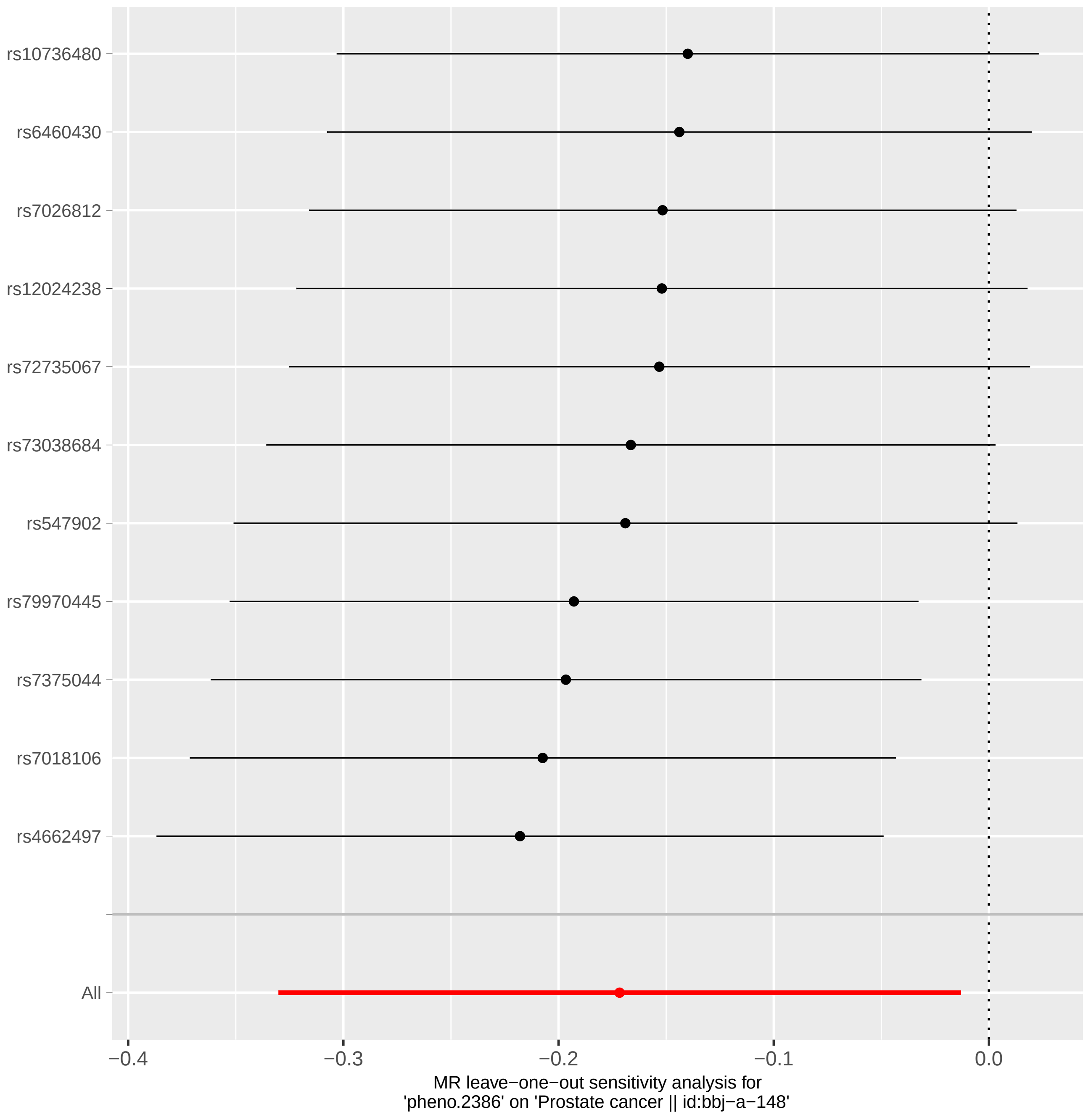
rs10736480
rs6460430
rs7026812
rs12024238
rs72735067
rs73038684
rs547902
rs79970445
rs7375044
rs7018106
rs4662497
All
−0.4
−0.3
−0.2
−0.1
0.0
MR leave−one−out sensitivity analysis for
'pheno.2386' on 'Prostate cancer || id:bbj−a−148'
